# Impact of Antiretroviral Therapy on Renal Function among HIV-Infected Tanzanian Adults: A Retrospective Cohort Study

**DOI:** 10.1371/journal.pone.0089573

**Published:** 2014-02-26

**Authors:** Bonaventura C. T. Mpondo, Samuel E. Kalluvya, Robert N. Peck, Rodrick Kabangila, Benson R. Kidenya, Lucheri Ephraim, Daniel W. Fitzgerald, Jennifer A. Downs

**Affiliations:** 1 Department of Medicine, Catholic University of Health and Allied Sciences, Mwanza, Tanzania; 2 Department of Medicine, Bugando Medical Centre, Mwanza, Tanzania; 3 Department of Medicine, Weill Cornell Medical College, New York, New York, United States of America; 4 Department of Biochemistry and Molecular Biology, Catholic University of Health and Allied Sciences, Mwanza, Tanzania; University of Texas Health Science Center San Antonio Texas, United States of America

## Abstract

**Background:**

Data regarding the outcomes of HIV-infected adults with baseline renal dysfunction who start antiretroviral therapy are conflicting.

**Methods:**

We followed up a previously-published cohort of HIV-infected adult outpatients in northwest Tanzania who had high prevalence of renal dysfunction at the time of starting antiretroviral therapy (between November 2009 and February 2010). Patients had serum creatinine, proteinuria, microalbuminuria, and CD4^+^ T-cell count measured at the time of antiretroviral therapy initiation and at follow-up. We used the adjusted Cockroft-Gault equation to calculate estimated glomerular filtration rates (eGFRs).

**Results:**

In this cohort of 171 adults who had taken antiretroviral therapy for a median of two years, the prevalence of renal dysfunction (eGFR <90 mL/min/1.73 m^2^) decreased from 131/171 (76.6%) at the time of ART initiation to 50/171 (29.2%) at the time of follow-up (p<0.001). Moderate dysfunction (eGFR<60 mL/min/1.73 m^2^) decreased from 21.1% at antiretroviral therapy initiation to 1.1% at follow-up (p<0.001), as did the prevalence of microalbuminuria (72% to 44%, p<0.001). Use of tenofovir was not associated with renal dysfunction at follow-up.

**Conclusion:**

Mild and moderate renal dysfunction were common in this cohort of HIV-infected adults initiating antiretroviral therapy, and both significantly improved after a median follow-up time of 2 years. Our work supports the renal safety of antiretroviral therapy in African adults with mild-moderate renal dysfunction, suggesting that these regimens do not lead to renal damage in the majority of patients and that they may even improve renal function in patients with mild to moderate renal dysfunction.

## Introduction

Major advances in HIV care, particularly the widespread implementation of antiretroviral therapy (ART), have improved survival and decreased the incidence of opportunistic infections worldwide [1]. With these advances, non-infectious causes of mortality such as cardiovascular, liver, and kidney disease have become more common sources of morbidity and mortality among HIV-infected patients in both high- and low-income countries [2].

Studies from several countries in sub-Saharan Africa have demonstrated a high prevalence of renal dysfunction in HIV-infected individuals, reporting that 34% to 77% of patients have estimated glomerular filtration rates (eGFRs) <90 ml/min/1.73 m^2^ [3]–[6]. At our hospital, we recently found that 64% of HIV-infected patients had decreased eGFRs at the time of beginning ART, while 72% had microalbuminuria [7]. In addition, 25% of our patients had eGFRs <60 ml/min/1.73 m^2^ and were therefore at increased risk for ART-related side effects. These findings were concerning given reports that renal dysfunction is associated with increased hazard of death among HIV-infected patients initiating ART [4], [8].

Although renal dysfunction is known to be common in this population, data regarding the progression of renal dysfunction after the initiation of ART are conflicting. Several studies have followed patients for up to 12 years and demonstrated that baseline renal dysfunction improves after ART initiation in HIV-infected patients, regardless of which ART regimen is used [9]–[17]. Others have reported no significant improvement and/or ongoing renal decline with similar follow-up periods, regardless of etiology of renal dysfunction or virologic suppression [18]–[21], though virologic suppression did appear to attenuate the rate of decline [20]. We hypothesized that the use of ART would improve renal status in a cohort of HIV-infected Tanzanian adults who had been found to have high rates of mild or moderate renal dysfunction at the time of ART initiation. In contrast to other studies that have yielded conflicting results, we began with a cohort of patients with a high burden of baseline renal dysfunction in a setting in which tenofovir-based regimens were being widely used. We also aimed to follow patients for a longer period of time, and therefore conducted a follow-up study to evaluate renal function in this cohort of outpatients a median of 2 years after initiating ART.

## Materials and Methods

### Ethics Statement

Patients had consented for initial participation in the study done at the time of ART initiation and provided a second written informed consent prior to enrollment in this follow-up study. This study was approved by the institutional review boards of both the Catholic University of Health and Allied Sciences (CUHAS)/BMC and Weill Cornell Medical College.

### Study Design and Participants

This study was conducted among adults receiving care at the HIV outpatient clinic at Bugando Medical Centre (BMC). BMC is the referral hospital for 13 million people in northwest Tanzania. It is located on the shore of Lake Victoria where the prevalence of HIV is over 6% [22]. BMC’s HIV clinic serves ∼12,000 patients, with over 3,500 on ART. A subset of 238 HIV-infected adults from the original cohort who were registered for primary care at BMC were sought for additional study participation. These patients had been previously screened for renal dysfunction when they were initiating ART between September 2009 and February 2010 [7]. Among these 238 patients, 17/238 had died, 32/238 had been lost to follow-up and 18/238 did not consent to additional participation.

### Data Collection and Laboratory Analyses

A structured questionnaire was used to collect demographic information. Baseline data (at the time of ART initiation) including CD4^+^ T-cell count (CD4 count), body mass index (BMI), serum creatinine, proteinuria, and microalbuminuria were obtained from the HIV clinic database. These measurements were repeated at the time of follow-up using the same reagents and laboratory instruments used at baseline. Serum creatinine was measured using a Cobas 400 clinical chemistry machine (Roche, Germany), calibrated by the IDMS-traceable Creatinine Jaffe 2 method. Spot proteinuria and microalbuminuria were measured in urine specimens using rapid test strips (Multistix 10SG (Siemens, USA) and Micral-TestB (Boehringer Mannheim, Germany), respectively.

### Statistical Analysis

Continuous variables were summarized by medians and interquartile ranges (IQRs) and categorical variables were summarized by frequencies and percentages. We used.

Pearson’s Chi square to compare categorical variables and paired t-tests and Wilcoxon signed-rank tests to compare continuous variables at ART initiation and at follow-up. Wilcoxon rank-sum tests were used to compare eGFRs between groups of patients with different outcomes. Univariable followed by multivariable logistic regression were used to determine the association between baseline characteristics and outcomes (renal dysfunction at follow-up, death, loss-to-follow-up), and were summarized with odds ratios (ORs) and 95% confidence intervals (CIs) with associated p-values. Two-way hypotheses/confidence intervals were used for all calculations. Data were analyzed using StataIC/11.1 (College Station, Texas). Estimated glomerular filtration rates (eGFRs) were calculated by the Cockroft-Gault (CG) equation as a primary outcome since the CG formula had been used in analysis of the baseline cohort. We adjusted the creatinine clearance by CG for a body surface area of 1.73 m^2^ using the Mostellar formula [23], as had been done at baseline. We secondarily calculated eGFRs by the abbreviated Modification of Diet in Renal Disease (MDRD) equation, as had been done with the original cohort. For simplicity, all eGFR values reported in the remainder of the manuscript can be assumed to have units of mL/min/1.73 m^2^.

## Results

### Patient Characteristics

Two hundred thirty-eight patients were enrolled from November 2009 to February 2010 and followed for a median of two years. The median age was 38 (IQR, 33–44) years and ∼70% were female. Over 90% of patients had no more than primary schooling, and over 80% were either unemployed or self-employed. Nearly two-thirds of patients lived with their partners (either married or cohabiting).

Median baseline CD4 count at the time of starting ART was 143 (78–187) cells/µL, and median eGFR was 72.7 (55.1–100.7) by CG and 93.0 (66.0–129.0) by MDRD. Among these 238 patients, 153 (64.3%) had baseline decreased eGFR <90 by CG and 116 (48.7%) by MDRD. The most common ART regimens used were zidovudine/lamivudine/efavirenz (30%), stavudine/lamivudine/nevirapine (25%), tenofovir/emtricitabine/efavirenz (23%), and zidovudine/lamivudine/nevirapine (22%), and 169/171 patients were taking cotrimoxazole at the time of follow-up. Of note, 8 of the 40 patients who were treated with a tenofovir-containing regimen had a baseline eGFR <60 by CG.

### Clinical and Laboratory Characteristics of Study Patients After Two Years on ART

At the time of follow-up, 17 patients had died, 32 were lost to follow-up, and 18 did not consent to additional testing. Among the remaining 171 patients, the median time on ART was 2.0 (1.8–2.4) years. All except two patients were currently receiving ART. Most patients experienced increases in both CD4 count and BMI, with the median CD4 count rising from 143 to 396 cells/µl and the BMI rising from 21 to 24.3 kg/m^2^. Nine patients met criteria for World Health Organization (WHO) immunological failure with CD4 counts either persistently <100 (3 patients) and/or below baseline (8 patients).

### Predictors of Death or Loss to Follow-up

The baseline median eGFR among the 49 patients who ultimately died or were lost to follow-up was 65.6 (51.8–96.6), which was not statistically different from the baseline eGFRs of the 171 who participated or the 18 who declined to participate (eGFR = 74.1 (55.8–100.1), p = 0.20 and eGFR = 70.7 (58.5–112.3), p = 0.29 respectively, by Wilcoxon rank-sum). On multivariable logistic regression analysis, factors that were significantly associated with death or loss to follow-up included less education (OR 0.10 for each increasing increment of education [0.04–0.23], p<0.001), living with one’s partner (married or cohabiting, OR = 4.0 [1.5–10.2], p = 0.004), and lower baseline BMI (OR = 0.89 for each increasing kg/m^2^ [0.80–0.99], p = 0.033). These factors remained significant when analyzed for death alone (n = 17) without the other 32 patients who were lost to follow-up.

Baseline decreased eGFR (<90 by CG) demonstrated a borderline trend towards association with death (OR = 2.8 [0.8–9.9], p = 0.12) though not loss to follow-up (OR = 1.3 [0.6–2.8], p = 0.57). Baseline decreased eGFR by MDRD was not associated with death or loss to follow-up. Neither baseline nor current low eGFR (<90 by CG) was associated with immunological treatment failure (p = 0.47 and p = 0.74, respectively). Among the 54 patients who were originally treated with tenofovir, 5 died (9.3%) compared with 12 of the 184 patients (6.5%) who were not treated with tenofovir (p = 0.55).

### Renal Outcomes

At the time of follow-up, patients’ eGFRs by CG equation had improved, from a median of 74.1 (55.8–100.1) at baseline to 103.4 (85.3–135.6) at follow-up (p<0.001) (**Table 1** **).** At follow-up, 36 of 171 (21.1%) had decreased eGFRs of <90, and only 2 (1.2%) had eGFRs <60 compared to 107/171 (62.6%) and 36/171 (21.1%) respectively at baseline (p<0.001 for each). By MDRD, 23 of 171 (13.5%) had eGFRs <90, and the same two patients had eGFRs <60. The prevalence of microalbuminuria decreased from 72.1% to 43.9% (p<0.001), and the prevalence of proteinuria decreased from 35.7% to 8.8% (p<0.001) at follow-up. All eight patients who had had initial eGFRs <60 and were treated with tenofovir had follow-up eGFRs >60 and stable (n = 3) or improved (n = 5) microalbuminuria and proteinuria.

**Table 1:** Pre- and post-treatment renal outcomes in 171 HIV-infected patients on ART followed up after a median of 2 years at Bugando Medical Centre.

Characteristic	Value At ART Initiation [Number(percent) or Median (IQR)]	Value At Follow-up [Number(percent) or Median (IQR)]	p-value
eGFR by CG equation (mL/min/1.73 m^2^)	74.1 (55.8–100.1)	103.4 (85.3–135.6)	<0.001
eGFR by MDRD equation (mL/min/1.73 m^2^)	87.5 (64.3–125.3)	125.1 (103.5–152.8)	<0.001
CKD Stage by CG equation			
Stage 0 (eGFR >90)	40 (23.4)	121 (70.8)	<0.001
Stage 1 (eGFR <90 with proteinuria)	24 (14.0)	14 (8.2)	
Stage 2 (eGFR 60–89)	71 (41.5)	34 (19.9)	
Stage 3 (eGFR 30–59)	36 (21.1)	2 (1.2)	
Stage 4 (eGFR 15–29)	0	0	
Stage 5 (eGFR <15)	0	0	
CKD Stage by MDRD equation			
Stage 0 (eGFR >90)	55 (32.2)	133 (77.8)	<0.001
Stage 1 (eGFR <90 with proteinuria)	28 (16.4)	15 (8.8)	
Stage 2 (eGFR 60–89)	57 (33.3)	21 (12.3)	
Stage 3 (eGFR 30–59)	31 (18.1)	2 (1.2)	
Stage 4 (eGFR 15–29)	0	0	
Stage 5 (eGFR <15)	0	0	
Serum creatinine (µmol/L)	81.0 (62.8–105.3)	60.9 (50.0–71.4)	<0.001
Microalbuminuria (>20 mg/dL)	123 (71.9%)	74 (43.5%)	<0.001
Proteinuria	61 (35.7%)	15 (8.8%)	<0.001

Non-missing data are included in each calculation.

### Factors Associated with Decreased eGFR on ART

By univariable logistic regression analysis, decreased eGFR (<90 by CG) after an average of two years on ART was significantly associated with following baseline characteristics: lower CD4 count (OR 0.995 for each cell/µL increase [0.99–0.999], p = 0.030), lower BMI (OR 0.9 for each unit increase [0.8–0.99], p = 0.036), and older age (OR 1.06 for each additional year of age [1.02–1.1], p = 0.004). When subjected to multivariable analysis, age, baseline CD4 count and baseline BMI remained significantly associated with decreased eGFR (**Table 2**).

**Table 2:** Univariable and Multivariable Analyses of Baseline Predictors of Decreased Renal Function (eGFR <90 mL/min/1.73 m^2^) after a Median of 2 years of Antiretroviral Therapy (n = 171).

Univariable Multivariable						
Variable	Number (%) ormedian (IQR)with eGFR<90(n = 36)	Number (%) ormedian (IQR) witheGFR>90 (n = 135)	OR [95% CI]	P value	OR [95% CI]	P value
Age at ART initiation (yrs)	43 (38–48)	38 (33–43)	1.06 [1.02–1.1]	0.004	1.06 [1.01–1.11]	0.009
Female sex	23 (63.9)	98 (72.6)	1.5 [0.7–3.3]	0.31		
Education						
Uneducated	10 (27.8)	27 (20.0)	0.86 [0.5–1.5]	0.59		
Primary	21 (58.3)	92 (68.2)				
Secondary	4 (11.1)	11 (8.2)				
College	1 (2.8)	5 (3.7)				
Unemployed or petty trader	28 (77.8)	109 (80.7)	0.83 [0.3–2.0]	0.69		
Living alone	17 (47.2)	58 (43.0)	1.2 [0.6–2.5]	0.64		
Baseline CD4 (cells/µl)	115 (62–174)	159 (85–203)	0.995 [0.99–0.999]	0.030	0.99 [0.989–0.999]	0.015
Baseline BMI (kg/m2)	21 (19–22.5)	21 (19–24)	0.9 [0.8–0.99]	0.036	0.88 [0.78–0.998]	0.047
Baseline serum creatinine(umol/l)	72 (59–104)	82(64–105)	0.99 [0.98–1.01]	0.30		
Baseline eGFR (ml/min/1.73 m2)	87.9 (62.6–115.5)	80.6 (63.5–105.1)	1.0 [0.99–1.01]	0.75		
Received tenofovir-basedbaseline ART regimen	6 (16.7)	34 (25.2)	0.59 [0.2–1.5]	0.29		

Of note, neither baseline decreased eGFR nor tenofovir use was associated with decreased eGFR at follow-up. In fact, we observed a trend towards renal improvement in those who received tenofovir-containing regimens (OR for odds of improvement = 1.97 [0.91–4.3], p = 0.09). In addition, each subsequent year on ART was associated with a trend towards protection against decreased eGFR (OR = 0.6 [0.4–1.1]. p = 0.13).

## Discussion

This study, which followed a cohort HIV-infected patients in whom nearly two-thirds had renal dysfunction at the time of initiating ART, revealed substantial renal improvement in the majority of patients after a median of two years. The prevalence of renal dysfunction dropped from 77% pre-ART to 29% at follow-up, and the prevalence of moderate renal dysfunction (eGFR <60) dropped from 21% to 1%. We additionally noted significant decreases in the prevalence of microalbuminuria and proteinuria (72% to 44% and 36% to 9%, respectively). In the face of conflicting reports on the utility of ART for reversing renal dysfunction, our findings provide reassurance that, even in the setting of mild-moderate renal dysfunction, ART is safe and is more likely to improve than to worsen renal function.

These findings have particular import in sub-Saharan Africa, where the burden of renal disease among HIV-infected patients is high [5]–[7], [24], and where routine monitoring for renal toxicity is often either not available or cost-prohibitive. Predictors of decreased eGFR after two years of ART included older age, lower BMI, and lower CD4 count but, notably, did not include the patient’s baseline eGFR or whether the patient received a tenofovir-containing regimen. In fact, our data showed a non-significant trend towards renal improvement in those who received tenofovir-containing regimens. Our findings support the WHO’s 2013 recommendation for a tenofovir-based first-line ART regimen and provide reassurance that, in most patients, tenofovir treatment likely does not require renal monitoring for toxicity [25].

Our work extends the findings of other studies conducted in sub-Saharan Africa. A study in rural Uganda similarly reported improvement in renal dysfunction of most HIV-infected patients after ART initiation [9]. In a Ghanaian retrospective study, 44% of ART-naïve patients had eGFRs <60, compared with 15% of those on ART [4]. Notably, ART regimens did not include tenofovir for patients in either of these studies. The DART study, conducted in Uganda and Zimbabwe, reported improvement in eGFR regardless of the type of ART used, including among the 74% of patients who received tenofovir plus co-formulated zidovudine-lamivudine [10]. Median baseline CD4 counts of patients in each of these studies ranged from 86–133, which is comparable to our patients’ median of 143 cells/µL.

Furthermore, our data supports the safety of tenofovir use in sub-Saharan Africa. A 2010 meta-analysis found that tenofovir was associated with a significant, though clinically-modest, loss of renal function [26]. Importantly, none of the 17 studies included in the meta-analysis were conducted in sub-Saharan Africa and only a portion of one study was conducted outside of the US, Europe, Australia, or Japan. Studies to assess the safety of tenofovir in sub-Saharan Africa have yielded conflicting reports, leading to concerns about utilization of tenofovir in resource-limited settings [27]–[29]. Baseline CD4 counts in these African studies were comparable to or slightly higher than those in our study, with the highest median CD4 count at 209 cells/µL [28].

Our study has several limitations. First, like the other studies of renal function and tenofovir safety in sub-Saharan Africa, this is an observational cohort study and not a randomized clinical trial. An additional limitation of our study, as with many studies of HIV in sub-Saharan Africa, is that we were not able to measure viral loads. These limitations highlight the salient role that a rigorously-conducted randomized controlled trial in this setting would play in clarifying this issue.

In conclusion, the remarkably high prevalence of renal dysfunction in this cohort of HIV-infected adults who began ART in Tanzania in 2009 had improved in the large majority of patients when reassessed after ∼2 years of ART. Our data also provide some additional support for the WHO’s recommendation for tenofovir-based regimens in sub-Saharan Africa. Though our sample size was small, we found that patients given tenofovir-containing regimens experienced renal stability or improvement, even if they had pre-existing mild to moderate renal dysfunction. We cautiously support use of tenofovir without routine renal monitoring in patients initiating ART in resource-limited settings such as sub-Saharan Africa, although larger studies are needed to confirm our findings.
